# Dr. Henry L. Elsner

**Published:** 1916-04

**Authors:** 


**Dr. Henry L. Elsner,** of Syracuse, died suddenly on the afternoon of February 17, 1916, in Washington, D. C. His death was the final phase of a chronic cardio-vascular condition which he had known and which had caused apprehension amongst his intimate friends for several years.

Dr. Elsner was born in Syracuse, N. Y., August 15, 1857. His father was a physician and the son never thought of any other career for himself. His education preliminary to medicine was carried on in the public schools of his native city, from which he went to the College of Physicians and Surgeons, now the Medical Department of Columbia University, and from it received the degree of M. D. in 1877. That same year his cousin, the late Dr. Nathan Jacobson, was graduated from the Syracuse Medical College. Together they journeyed in the autumn to Vienna and for a year were under the instruction of the eminent professors of the Vienna University Medical School, a faculty at that time of the highest repute in the world. Dr. Elsner selected work that should fit him as an internist, while Dr. Jacobson chose the field of surgery. They returned to Syracuse in 1879 and Dr. Elsner opened an office in North Salina Street. He attracted the attention of the best men in medicine very early and in 1882 he was nominated by Dr. H. D. Didima, Professor of the Theory and Practice of Medicine in the Syracuse Medical College as instructor in Medicine and was unanimously elected to that position. From that time until his death he was actively engaged in teaching. In 1884 he was advanced to the position of Lecturer, and in 1886 to the position of Professor of Clinical Medicine. Upon the retirement of Professor Didima from the deanship and from the chair in medicine in 1893, Dr. Elsner was elected full Professor of the Science and Art of Medicine.

In 1881 Dr. Elsner married Pauline Rosenberg of Rochester, a woman of great personal charm, keen intellect, and noble ideals. She was his comrade and his constant inspiration. Their beautiful life of closest companionship ended almost simultaneously. Mrs. Elsner nerved herself to endure the strain of her husband's death and directed the details of the funeral and followed his body to the tomb. She then collapsed and died in coma February 23.

Dr. Elsner was lavishly endowed by nature. He had a noble head and a fine manly body. His personality was charming. He was kind hearted and friendly with all. His disposition was sunny, and his manners cultured but democratic. He was animated by a noble purpose and had himself in perfect control under all the circumstances of life. He had accurate and rapid powers of observation, good judgment, a fine imagination, a remarkably retentive memory, and an innate gift of expression, which, under cultivation, made it easy for him to deliver an opinion with an effectiveness that none can forget. These qualities made him a natural leader in his profession and attracted and held a clientele of the most intelligent people.

He began to write out and present his observations on medical subjects early in his career, and the list of his contributions to medical literature is very long. In Forcheimer's Therapeusis of Internal Diseases he wrote the treatise on Pneumonia. At the solicitation of the Appletons he had just finished a work on Prognosis for their Monographic Medicine. This volume is about to be issued. It contains twelve hundred pages and is a treatise on Medicine with special attention directed to prognosis. This is the first serious work on Prognosis published in English. It reveals Dr. Elsner's great learning and the wealth of his extensive experience in internal diseases in a most masterly way. It is a worthy companion to the volumes on Clinical Diagnosis by Lewellys F. Barker, on Differential Diagnosis by M. Howard Fussell and on Special Pathology by Albion W. Hewlett.

Dr. Elsner received every honor which the medical profession could bestow upon a fellow member. He was elected successively to the presidency of the Syracuse Academy of Medicine, the Onondaga Medical Society, the Central New York Medical Society, the New York State Medical Society, to the House of Delegates and to the Committee on Education of the American Medical Association and to membership in the American Climatological and Clinical Society. In June, 1915, Syracuse University gave him the honorary degree of LL. D.

The teachers and students of the College of Medicine in which his best work was done can never forget him. He was accurate, painstaking, kindly, always ready with helpful suggestions and always willing to forward the interests of the school and of the students. As a consultant Dr. Elsner was very widely esteemed. His large and varied practice, his wide acquaintance with medical literature and with leaders of medical thought and practice, added to his keen power of observation, made his diagnosis certain and his counsel wise. It is not given to all of us to attain such a height, but we shall miss the lesson of his life if we fail to see that accurate diagnostic ability can proceed only from systematizing careful, painstaking and accurate observations of the phenomena of disease, and that the art of medicine is acquired only by him who is its constant student.—John L. Heffron.

